# Impact of ABO Incompatibility on the Development of Acute Antibody-Mediated Rejection in Kidney Transplant Recipients Presensitized to HLA

**DOI:** 10.1371/journal.pone.0123638

**Published:** 2015-04-21

**Authors:** Byung Ha Chung, Yu Young Joo, Jaesin Lee, Hyung Duk Kim, Ji-Il Kim, In Sung Moon, Bum Soon Choi, Eun-Jee Oh, Cheol Whee Park, Yong-Soo Kim, Chul Woo Yang

**Affiliations:** 1 Transplant research center, Seoul St. Mary's Hospital, College of Medicine, The Catholic University of Korea, Seoul, Korea; 2 Division of Nephrology, Department of Internal Medicine, Seoul St. Mary's Hospital, College of Medicine, The Catholic University of Korea, Seoul, Korea; 3 Department of Surgery, Seoul St. Mary's Hospital, College of Medicine, The Catholic University of Korea, Seoul, Korea; 4 Deparment of Laboratory Medicine, Seoul St. Mary's Hospital, College of Medicine, The Catholic University of Korea, Seoul, Korea; University of Toledo, UNITED STATES

## Abstract

Whether the coexistence of anti-A/B antibody and donor specific anti-HLA antibody (HLA-DSA) has a synergistic impact on the development of acute antibody-mediated rejection (AAMR) in kidney transplant recipients (KTRs) is unclear. This study includes 92 KTRs who received a kidney from an ABO-incompatible (ABOi) donor or were presensitized to donor HLA (HLAs) and 292 controls (CONT). HLAs was defined as a crossmatch positivity or the presence of HLA-DSA. We compared the incidence of AAMR among ABOi (n = 58), ABOi+HLAs (n = 12), HLAs (n = 22), and CONT (n = 292) groups and evaluated the risk factors and antibody type (anti-A/B vs. HLA-DSA) responsible for AAMR. AAMR developed less frequently in ABOi and CONT than in the ABOi+HLAs or HLAs (P < 0.05 for all); however, there was no difference between the ABOi+HLAs and HLAs groups. AAMR developed more frequently with strong HLA-DSA at baseline; however, high baseline anti-A/B titer did not affect AAMR development. Strong baseline HLA-DSA was an independent predictor for AAMR, however the baseline anti-A/B titer was not. All four AAMR episodes in ABOi+HLAs were positive to HLA-DSA but not to anti-A/B. In conclusion, ABO incompatibility does not increase the risk for AAMR in HLAs KTRs.

## Introduction

Both humoral sensitization to antigens of the human leukocyte antigen (HLAs) and ABO incompatibility (ABOi) have been important immunologic barriers to successful kidney transplantations (KTs) [1,2]. These two conditions have many similarities in treatment and clinical course. For example, a desensitization protocol comprising plasmapheresis (PP), intravenous immunoglobulin (IVIg), and rituximab (RTX) has been used in both conditions; moreover, in both, inadequate removal of the preformed antibody or development of a *de novo* antibody after KT may cause antibody-mediated tissue injury that can limit the long-term outcome of the allograft [3,4,5,6,7,8].

However, the two conditions have shown some differences in outcome. For example, many recent studies showed that the outcome of ABOi KT is comparable to ABO-compatible KT [1,4,7,9,10]. In contrast, KT recipients with presensitization to donor HLA still showed inferior clinical outcome in terms of acute rejection and allograft survival rate [4,11,12]. The reason for this is unclear; however, the difference in nature between the donor-specific anti-HLA antibody (HLA-DSA) and the anti-A/B antibody may result in the above-mentioned discrepant outcome between the two conditions.

In case of KT in combined ABOi and HLAs, it is still unclear whether the coexistence of both antibodies has a synergistic impact on the immunologic risk compared with KT in HLAs. Indeed, it has been rarely investigated and only a few published data exist about this issue [13,14,15,16]. In this study, we described our experiences of KT in recipients with combined ABOi and HLAs, and compared their outcomes with those of solely ABOi or HLAs KT recipients. Finally, we investigated whether ABOi affects the immunologic risk of patients presensitized to donor HLA.

## Patients and Methods

### Study population

**Fig 1:**
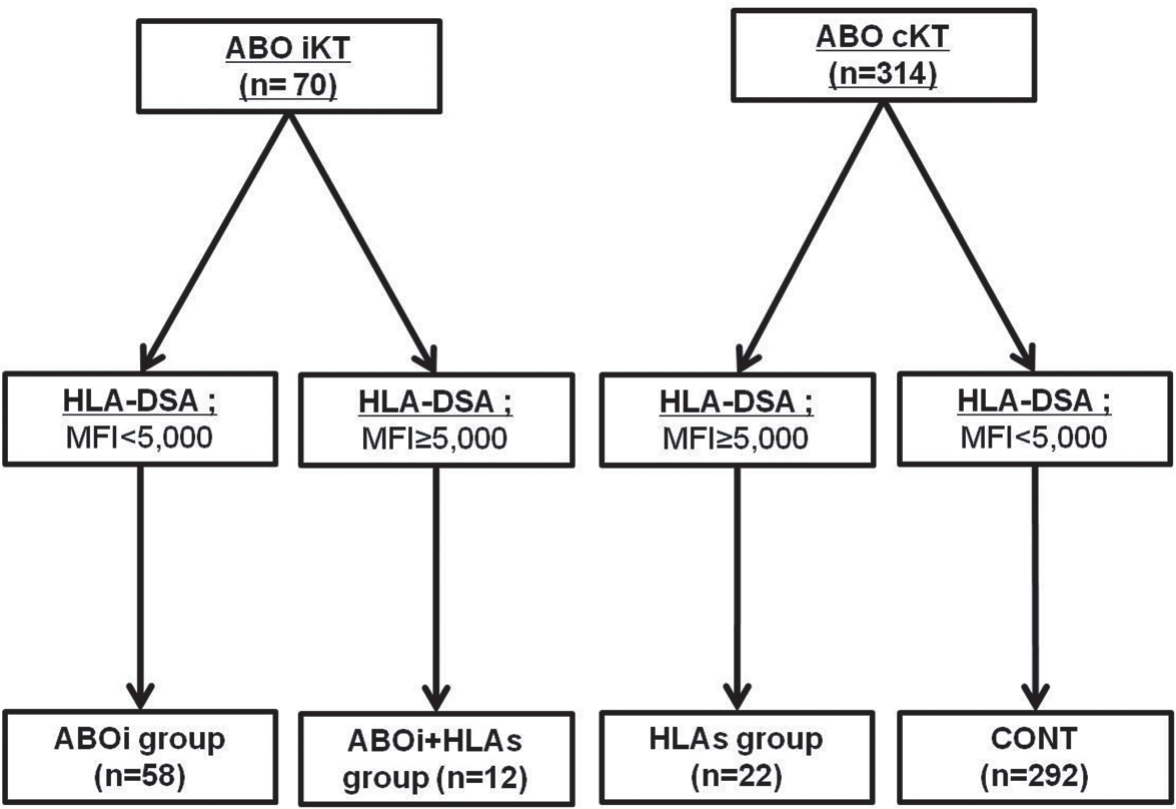
Distribution of the patient population according to ABO incompatibility to donor and immunologic risk. Of 384 patients screened, 70 cases were ABO incompatible KT, and another 314 cases were ABO compatible KT. Out of ABO incompatible KT, patients with negative or weak HLA-DSA (MFI<5,000) belonged to ABOi group and patients with moderate to strong HLA-DSA (MFI ≥ 5,000) were regarded as ABOi+HLAs group. Likewise, out of 314 ABO compatible KT, patients with moderate to strong HLA-DSA belonged to the HLAs group and patients with negative or weak HLA-DSA were defined as the control (CONT) group. AAMR, acute antibody mediated rejection; AR, acute rejection; ABOi, ABO incompatible; HLAs, sensitized to HLA; HLA-DSA, donor-specific anti-HLA antibody; CONT, control group; KT, kidney transplantation; MFI, median fluorescent intensity

From May 2009 to November 2013, 386 cases of living-donor KTs were performed in Seoul St. Mary’s Hospital (Seoul, Korea). We excluded 2 cases who took kidney and hematopoietic stem cell transplantation simultaneously; hence 384 cases were included. None of the transplant donors were from a vulnerable population and all donors or next of kin provided written informed consent that was freely given. In this study, ABOi means kidney transplantation from ABO incompatible donor and HLAs was defined as a positive result in any type of crossmatch test (complement-dependent cytotoxicity [T and B cell] or flow cytometric crossmatch [T and B cell]), or the presence of HLA-DSA with a median fluorescent intensity (MFI) value of >5000 in the Luminex single-antigen assay (LSA) (Tepnel Lifecodes Corp., Stamford, CT). According to above criteria, we divided patient population into four groups: ABOi (n = 58), ABOi+HLAs (n = 12), HLAs (n = 22) and control (CONT) (n = 292) (Fig 1). This study was approved by the institutional review board of Seoul St. Mary’s Hospital (KC11RCMI0716). Informed consent was waived because this study was done by retrospective medical record review. Patient records and information was anonymized and de-identified prior to analysis.

### Pretransplant immunologic testing

The immunologic work-up was performed as described previously [4]. Briefly, immunologic tests including crossmatching, panel-reactive antibody (PRA) screening and HLA typing were performed before KT. PRA screening test was done by the Luminex method (Lifecodes LifeScreen Deluxe kits; Hologic Gen-Probe Inc., San Diego, CA) and was presented as %PRA; CDC-XM and FCXM testing were performed in the standard manner [17,18]. HLA typing was performed using LIFECODES HLA-A, B, C, DRB1, DQB1 SSO Typing Kit (Immucor Transplant Diagnostics, Inc. 550 West Avenue, Stamford, CT 06902). This procedure was based on the hybridization of labeled single stranded PCR product to SSO probes.

When the PRA test was positive, we checked the presence of the anti-HLA antibody by using LSA. LSA assay for HLA-DSA was performed according to the manufacturer’s instructions, using Lifecodes LifeScreen Deluxe kits (Tepnel Lifecodes Corp., Stamford, CT) as described previously [4]. If the anti-HLA antibody detected in the serum of the patient corresponded to the HLA type of the donor, it was classified as HLA-DSA. The results were presented as MFI levels, and the recipients were classified into four groups according to their peak level at baseline: strong, >10,000; moderate, 5000–10,000; weak, 1000–5000; and negative, <1000.

The anti-A/B antibody titers in ABOi KT recipients were monitored as described previously [7,9,10]. Briefly, we measured both anti-A/B IgG and IgM; however, we used IgG as the criterion to determine the pretransplant number of PP/IVIg and to decide whether to reach the target titer to allow KT. The anti-A/B antibody titer was measured every day from the start of PP/IVIg treatment. The target titer at transplantation and during the first 2 weeks after transplantation was ≤1:16.

### Maintenance Immune suppressant protocol

The typical immunosuppressive regimen in our center was described previously [8,19]. Briefly, the initial immunosuppressant was tacrolimus (Tac) or cyclosporin (CsA) in combination with mycophenolate mofetil (MMF) and prednisolone. Basiliximab was used as induction therapy at 2 hours before transplantation and on day 4 after transplantation. The initial dose of Tac was 0.16 mg/kg per day orally, and of CsA, 7 mg/kg orally. The target trough levels were 8–12 ng/mL during the first 3 months and 3–8 ng/mL thereafter for Tac and 150–300 ng/mL during the first 3 months and 50–100 ng/mL thereafter for CsA. In all patients, methylprednisolone (1 g/day) was administered by intravenous infusion on the day of transplantation and was tapered to prednisolone at 30 mg/day on day 4 after transplantation. The initial dose of MMF was 1.5 g/day and the dose was modified to minimize adverse effects such as diarrhea or leukopenia. we administered basiliximab induction therapy on the day of KT and at posttransplant day 4.

In ABOi, ABOi+HLAs and HLAs group, we used pre-transplant desensitization therapy as described below and initiated immunosuppressant treatment at 7 days before transplantation. In CONT group, we initiated immune suppressant treatment at 2 days prior to transplantation and in patients who showed PRA > 50%, but had no additional immunologic risk (ABO compatible donor; negative cross match and weak or negative HLA-DSA), we used RTX at a dose of 375 mg/m^2^ at 7 days before transplantation.

### Pretransplant desensitization protocol

The desensitization protocol for each situation was performed as described previously [4,7,9,10]. In ABOi KT recipients, we used RTX (MabThera; Genentech Inc., San Francisco, CA, USA) with a dose of 100 or 375 mg/m^2^ at 30 days before transplantation and administered PP/IVIg until the anti-A/B antibody level decreased to ≤1:16. In addition, in the HLAs group, we used RTX at a dose of 375 mg/m^2^ at 2–3 weeks before KT and administered PP/IVIg until the HLA-DSA decreased to a weak level (MFI <5000) and the crossmatch test became negative. In the ABOi+HLAs group, we used the same protocol as in ABOi KT; however, the target of desensitization was not only to reach an anti-A/B titer of 1:16 but also to decrease HLA-DSA to the weak level.

### Monitoring and prophylaxis for opportunistic infections after transplantation

Infection prophylaxis was done as described previously [4,7,8,20]. Briefly, all patients received fluconazole, bactrim, and valacyclovir from the start of immunosuppressant therapy until 6 months after transplantation. BK virus (BKV) and cytomegalovirus (CMV) were monitored as described previously [20]. Briefly, BKV and CMV were monitored at 1, 3, 6, 9, and 12 months after transplantation by using a real-time polymerase chain reaction (PCR) method. When the CMV viral copy number increased to >5000 copies/mL, we used ganciclovir as a preemptive therapy. When the serum BKV DNA copy number exceeded 1 × 10^4^ copies/mL, we withheld MMF and performed a follow-up real-time PCR for BKV.

### Diagnosis and management of acute rejection

An indication biopsy was performed in cases of allografts that showed a >20% increase in serum creatinine concentration. A 16-gauge biopsy gun was used under ultrasonic localization. Indirect immunofluorescence staining was performed by using monoclonal antibodies against complement protein C4d (Biogenesis, Poole, UK; dilution 1:50) for detecting C4d deposition. C4d positivity was defined as diffuse (>50%) and linear staining of peritubular capillaries. Histopathological diagnosis was made according to the revised Banff 2007 classification [21]. We concurrently checked the presence of HLA-DSA at the time of indication biopsy. In the ABOi or ABOi+HLAs group, we also checked the anti-A/B antibody titer during the indication biopsy.

### Comparison of the clinical outcome parameters

We retrospectively reviewed the medical records of all patients and collected baseline characteristics including age, sex, and the relationship between recipients and donors. The primary outcome of this study is the incidence of acute antibody-mediated rejection (AAMR). The secondary outcomes are the incidence of overall acute rejection; other complications such as infection or postoperative bleeding; and change of allograft function, allograft, and patient survival rate.

### Statistical analysis

Statistical analysis was performed by using SPSS software (version 15.0; SPSS Inc., Chicago, IL, USA). Data are presented as mean ± SD, or counts and percentages, depending on the data type. For continuous variables, means were compared by using Student’s *t*-test. For categorized variables, Pearson’s chi-square test and Fisher’s exact test were used. Binary logistic regression analysis was used to investigate the risk factors for the development of AAMR. Allograft and patient survival rates were calculated by using Kaplan-Meier analysis, and the log-rank method was used to compare the survival rate between the three groups. All tests were two-tailed, and the results were considered significant when the P value was <0.05.

## Results

### Baseline and clinical characteristics

The mean age of all patients was 44.3 ± 11.5 years, and 211 patients (57.6%) were men. The overall median follow-up period was 27.4 months (range, 6.5–58.8 months). The most common indication for transplantation was primary glomerulonephritis (29.2%), followed by diabetes mellitus (19.8%) and hypertension (10.2%). The baseline characteristics of each group are presented in Table 1. The proportion of male patients was higher in the ABOi group than in the ABOi+HLAs and HLAs groups (P < 0.01 for each). The follow-up duration was longer in the HLAs group than in the ABOi and CONT group. The primary renal disease did not differ among the three groups.

**Table 1:** Baseline and immunologic characteristics of the patient populations.

	ABOi KT (n = 58)	ABOi+HLAs (n = 12)	HLAs (n = 22)	CONT (n = 292)
**Age (year)**	44.6 ± 10.1	45.0 ± 10.5	46.1 ± 8.8	44.0 ± 12.0
**Male, n (%)**	42 (72.4) ^**†**^ ^,^ ^‡^ ^,^ ^**§**^	3 (25.0) *, ^**§**^	6 (27.3) *,^**§**^	170 (58.2) *, ^‡^
**F/U month**	24.8 ± 16.3 ^‡^	27.8 ± 12.2	31.3 ± 14.4 *, ^**§**^	24.5 ± 9.5 ^‡^
**PRD, n (%)**				
Chronic GN	21 (36.2)	4 (33.3)	8 (36.4)	84 (28.8)
DM	13 (22.4)	4 (33.3)	5 (22.7)	54 (18.5)
HTN	13 (24.5)	0	25 (19.4)	29 (9.9)
ADPKD	2 (3.4)	0 (0.0)	0 (0.0)	7 (2.4)
SLE	1	0 (0.0)	0 (0,0)	10 (3.4)
Unknown	15 (25.9)	4 (33.3)	5 (22.7)	108 (37.0)
**HLA mismatch**	3.8 ± 1.5 ^**§**^	3.3 ± 1.2	3.6 ± 1.5 ^**§**^	2.7 ± 1.9 * ^,^ ^‡^
**LRD, n (%)**	32 (55.2) ^**§**^	6 (50.0)	17 (77.3)	212 (72.6) *
**Re-transplant, n (%)**	7 (12.0) ^**†**^ ^,^ ^‡^	5 (41.7) *, ^**§**^	8 (36.3) ^**§**^	26 (8.9) ^**†**^ ^,^ ^‡^
**PRA >50%, n (%)**	8 (13.8) ^**†**^ ^,^ ^‡^	11 (91.7) *, ^**§**^	18 (81.8) *, ^**§**^	34 (11.6) ^**†**^ ^,^ ^‡^
**Positive XM, n (%)**	0 (0.0) ^**†**^ ^,^ ^‡^	10 (83.3) *, ^**§**^	19 (86.4) *, ^**§**^	0 (0.0) ^**†**^ ^,^ ^‡^
**RTX dose**	^**†**^ ^,^ ^‡^ ^,^ ^**§**^	^**§**^	* ^**, §**^	*, ^**†**^ ^,^ ^‡^
Not done	0	0	0	258 (88.4)
100 mg/m^2^, n (%)	37 (64)	0 (0)	0 (0)	0
375 mg/m^2^, n (%)	21 (36)	12 (100)	22 (100)	34 (11.6)
**Number of PP/IVIg**	6.0±3.4	7.0±2.8	5.5±2.2	0030

ABOi, ABO incompatible; HLAs, sensitized to HLA; CONT, control; F/U, follow up; GN, glomerulonephritis; DM, diabetes mellitus; HTN, hypertension; ADPKD, autosomal dominant polycystic kidney disease; SLE, systemic lupus nephritis; LRD, living related donor; PRA, panel reactive antibody; XM, crossmatch; RTX, rituximab; PP, plasmapheresis; IVIg, intravenous immunoglobulin, CONT, control

* P<0.05 vs. ABOi

^†^ P<0.05 vs. ABOi+HLAs

^‡^ P<0.05 vs. HLAs

^§^ P<0.05 vs. CONT.

### Comparison of immunologic characteristics and desensitization procedure

The proportion of living related donors was lower in ABOI group compared to CONT group and the HLA mismatch number was lower in CONT group than ABOi and HLAs group. (P < 0.05 for each) But it did not differ among ABOi, ABOI-HLAs and HLAs group. The proportion of second transplants and patients with PRA >50% was significantly higher in the ABOi+HLAs and HLAs groups than in the ABOi and CONT group (P < 0.05 for all). The proportion of a positive crossmatch at baseline was also significantly higher in the ABOi+HLAs and HLAs groups than in the ABOi and CONT group (P < 0.05 for all). However, no difference was detected between the ABOi+HLAs and HLAs groups in those parameters (P > 0.05 in all comparison). Low-dose RTX (100 mg/m^2^) was used in 64% of the ABOi group patients; however, all patients in the ABOi+HLAs and HLAs groups took a typical dose of RTX (375 mg/m^2^) (P < 0.05 vs. ABOi for both). In 11.6% of the CONT group, typical dose RTX was used because of high PRA. The mean number of pretransplant PP/IVIg sessions administered was 6.02 (range, 3–16), and it did not differ among the three groups (P > 0.05 for all comparisons)(Table 1).

### Comparison of AAMR development

**Fig 2:**
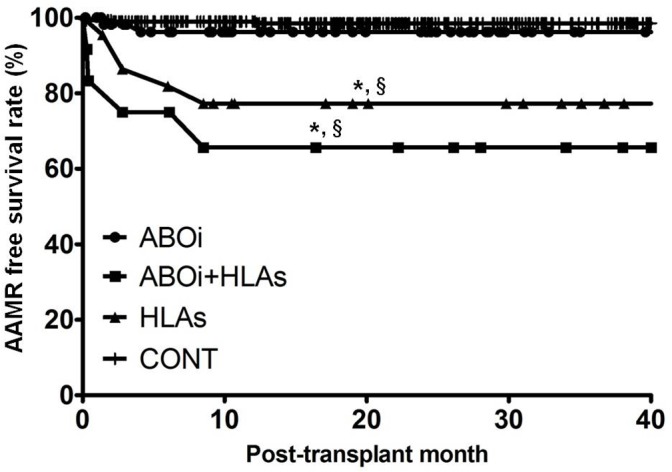
Comparison of the AAMR free survival rate among four groups. Note that AAMR free survival rate was significantly higher in the ABOi or CONT group compared to in the ABOi+HLAs or HLAs group. AAMR, acute antibody mediated rejection; ABOi, ABO incompatible; HLAs, sensitized to HLA; CONT, control group; HLA-DSA, donor-specific anti-HLA antibody. * P<0.05 vs. ABOi, ^†^ P<0.05 vs. ABOi+HLAs, ^‡^ P<0.05 vs. HLAs, ^§^ P<0.05 vs. CONT.

The incidence of AAMR episode per person was significantly lower in the ABOi or CONT group than in the ABOi+HLAs or HLAs group (P < 0.05 for all). AAMR free survival rate was significantly higher in the ABOi or CONT group compared to the other two groups as well (P < 0.05 for all)(Fig 2). The overall incidence of acute rejection episode per person was also significantly lower in the ABOi and CONT group than in the other two groups (P < 0.05 for all). However, there was no difference between the ABOi and CONT group or between the ABOi+HLAs and HLAs groups in those parameters (Table 2). In addition, we checked the HLA-DSA or anti A/B antibody to identify which antibody is associated with AAMR. In the HLAs group, HLA-DSA was detected in five of seven cases of AAMR (71.4%); it was also detected in all four AAMR cases from the ABOi+HLAs group. In contrast, anti-A/B antibody was negative in AAMR cases of the ABOi+HLAs group. In the two AAMR cases from ABOi group, rebound of anti-A/B antibody was found, but HLA-DSA was not detected (Table 3).

**Table 2:** Comparison of acute rejection episodes and other complications.

	ABOi KT (n = 58)	ABOi+HLAs (n = 12)	HLAs (n = 22)	CONT (n = 292)
**AAMR episodes, n (AAMR/person)**	2 (0.03)^†^ ^,^ ^‡^	4 (0.33)* ^,^ ^§^	8 (0.36)* ^,^ ^§^	4 (0.01) ^†^ ^,^ ^‡^
**Overall AR episodes, n (AR/person)**	12 (0.21)^†^ ^,^ ^‡^	13 (0.83)* ^,^ ^§^	18 (0.82)* ^,^ ^§^	40 (0.14) ^†^ ^,^ ^‡^
**Infection episodes, n (Infection/person)**	29 (0.50)	6 (0.50)	15 (0.68)	86 (0.40)
Bacterial infection episode, n (Infection/Person)	12 (0.20)	3 (0.25)	9 (0.40)	30 (0.10)
Viral infection episode, n, (Infection/person)	15 (0.26)	3 (0.25)	6 (0.27)	56 (0.19)
Fungal infection episode, n (Infection/Person)	2 (0.03)	0 (0)	0 (0)	0 (0)
**Post-operative bleeding, n (%)**	6 (10.3)	2 (16.7)	1 (4.5)	3 (1.0)* ^,^ ^†^

ABOi, ABO incompatible; HLAs, sensitized to HLA; CONT, control; RTX, rituximab; PP, plasmapheresis; IVIg, intravenous immunoglobulin; AR, acute rejection; AAMR, acute antibody mediated rejection

* P<0.05 vs. ABOi

^†^ P<0.05 vs. ABOi+HLAs

^‡^ P<0.05 vs. HLAs

^§^ P<0.05 vs. CONT.

**Table 3:** Anti-A/B antibody or HLA-DSA status at the time of antibody mediated rejection.

		Donor Recipient Blood type	Anti-A/B Antibody	HLA-DSA
**ABOi**	Case 1	A → O	Anti-A IgG > 1:1024	(-)
	Case 2	B → O	Anti-B IgG: 1:128	(-)
**ABOi+HLAs**	Case 3	AB → O	Anti-A IgG; 1:16, Anti-B IgG; 1:8	DR01, DR07
	Case 4	B → O	Anti-B IgG; Negative	A02, DR08
	Case 5	A → B	Anti-A IgG; Negative	DR09
	Case 6	AB → A	Anti-B IgG < 1:4	DR08
**HLAs**	Case 7	AB → AB	N/A	(-)
	Case 8	B → B	N/A	(-)
	Case 9	B → B	N/A	A11
	Case 10	O → AB	N/A	A29
	Case 11	A → A	N/A	DR10
	Case 12	A → A	N/A	DR09
	Case 13	O → O	N/A	B51
**CONT**	Case 14	A →A	N/A	(-)
	Case 15	B →B	N/A	DR08
	Case 16	O →O	N/A	DR53

ABOi, ABO incompatible; HLAs, sensitized to HLA; HLA-DSA, donor-specific anti-HLA antibody; CONT, control; N/A, non-applicable

### Prediction of AAMR by HLA-DSA or anti-A/B antibody titer at baseline

**Fig 3:**
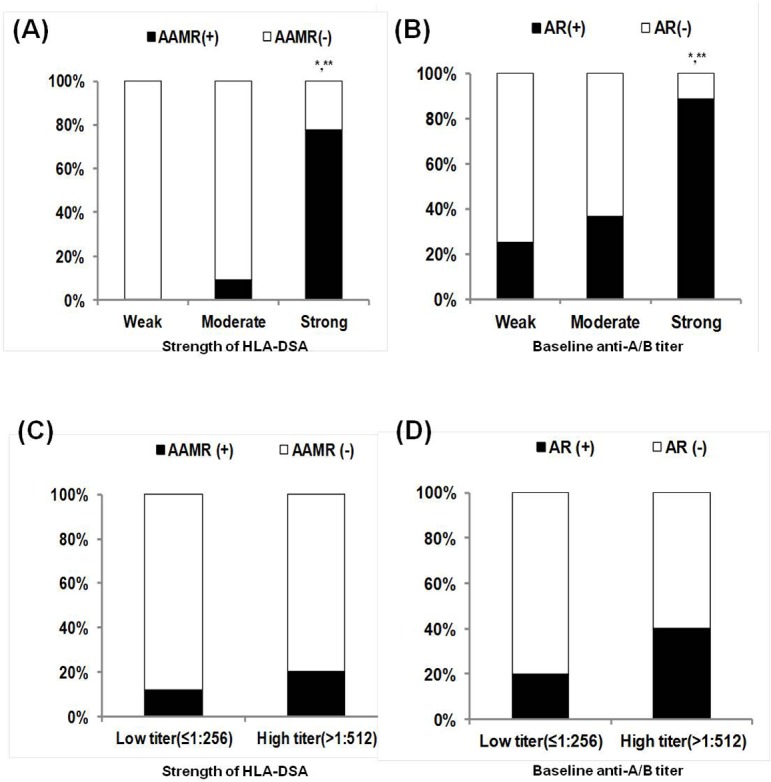
Comparison of the incidence of AAMR or overall acute rejection in subgroups divided according to baseline anti-A/B titer or HLA-DSA level at baseline. In analysis within ABOi+HLAs and HLAs group, prevalence of **(A)** AAMR and **(B)** overall AR was significantly higher in patients with strong HLA-DSA (MFI ≥ 10,000) at baseline compared to patient with negative to weak (MFI<5,000) or moderate HLA-DSA (MFI; 5,000~10,000) at baseline. However, in analysis within ABOi and ABOi+HLAs group, the prevalence of **(C)** AAMR and **(D)** overall AR did not show significant increase in patients with high baseline anti-AB antibody titer (≥1:512) compared to low titer group (≤1:256). AAMR, acute antibody mediated rejection; AR, acute rejection; ABOi, ABO incompatible; HLAs, sensitized to HLA; HLA-DSA, donor-specific anti-HLA antibody; MFI, median fluorescent intensity. * P<0.05 vs. Weak, ** P<0.05 vs Moderate

In our previous reports, high anti-A/B antibody titer (≥1:512) or strong HLA-DSA level (MFI ≥10,000) at baseline was a significant predictor for the development of acute rejection in ABOi or HLAs KT recipients, respectively [4,7]. We analyzed the association between the above factors and the AAMR episodes in this study population, including ABOi, HLAs and ABOi+HLAs KT recipients. In the analysis including only the ABOi+HLAs and HLAs groups (n = 34), a significant increase in AAMR and overall AR was seen in patients with a strong baseline HLA-DSA (7 of 9, 77.8%) compared with recipients with a moderate or weak HLA-DSA (P < 0.05 for both) (Fig 3A and 3B). However, in the analysis including only the ABOi+HLAs and ABOi groups (n = 70), no increase in AAMR was observed in patients with a high baseline anti-A/B antibody titer (2 of 10, 20%) compared with recipients with a low baseline anti-A/B titer (7 of 60, 11.7%) (P = 0.16)(Fig 3C). The overall rejection incidence shows similar results to the above analysis (Fig 3D). In multivariate analysis including the whole study cohort, a strong HLA-DSA at baseline was an independent risk factor for the development of AAMR; however, a high baseline anti-A/B titer was not (Table 4).

**Table 4:** Risk factors for the development of acute antibody mediated rejection.

	Univariate			Multivariate		
	HR	95% CI	*P*	HR	95% CI	*P*
**ABOi KT**	0.30	0.11–0.81	0.02	0.89	0.15–5.16	0.89
**ABOi+HLAs**	1.88	0.54–6.56	0.32	-	-	-
**HLAs**	3.80	1.48–9.72	<0.01	0.67	0.09–4.84	0.69
**High PRA (> 50%)**	3.04	1.21–7.70	0.02	0.93	0.18–4.68	0.93
**Donor type (LURD)**	1.28	0.52–3.18	0.59	-	-	-
**Re-transplant**	1.40	0.57–3.48	0.46	-	-	-
**HLA mismatch number**	0.90	0.66–1.23	0.51	-	-	-
**Anti-A/B antibody >1:512**	1.71	0.44–6.62	0.44	-	-	-
**Strong HLA-DSA**	35.4	4.16–301.58	<0.01	10.8	1.2–99.8	<0.01
**HLA-DSA-A**	18.8	4.8–73.8	<0.01	0.20	0.01–5.0	0.32
**HLA-DSA-B**	22.6	6.3–81.3	<0.01	4.7	0.43–50.6	0.21
**HLA-DSA-C**	26.3	1.56–442.2	0.02	8.7	0.03–2322.6	0.45
**HLA-DSA-DR**	52.9	14.5–193.5	<0.01	14.9	0.90–219.3	0.08
**HLA-DSA-DQ**	48.5	12.5–188.8	<0.01	5.3	0.56–50.8	0.15
**RTX use**	0.17	0.05–0.54	<0.01	1.0	1.00–1.02	0.15

ABOi, ABO incompatible; HLAs, sensitized to HLA; KT, kidney transplantation; PRA, panel reactive antibody; LURD, living unrelated donor; RTX, rituximab, HLA-DSA; donor-specific anti-HLA antibody

### Comparison of other complications, patient survival, and allograft survival

**Fig 4:**
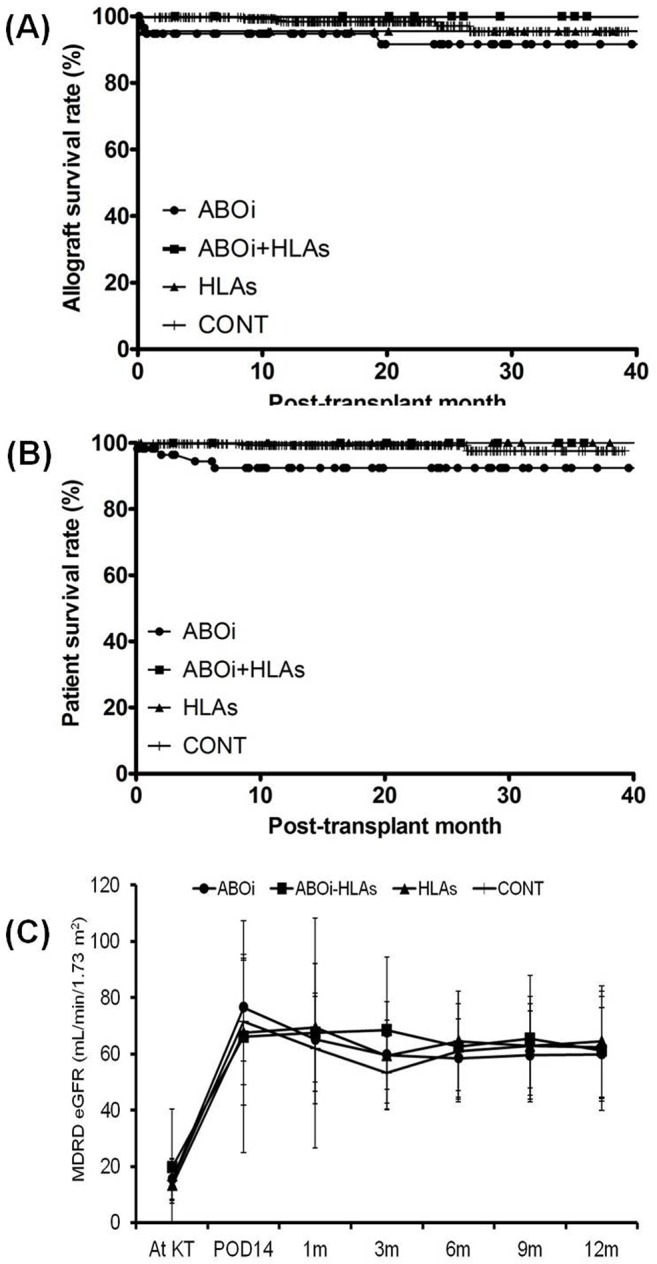
Comparison of allograft, patient survival rate and the change of allograft function. No difference was detected among four groups in (A) allograft survival rate, (B) patient survival rate and the (C) change of allograft function up-til 1 year from transplantation. ABOi, ABO incompatible; HLAs, highly sensitization to HLA; CONT, control group; MDRD; modification of diet in renal disease; eGFR, estimated glomerular filtration disease. * P<0.05 vs. ABOi, ^†^ P<0.05 vs. ABOi+HLAs, ^‡^ P<0.05 vs. HLAs, ^§^ P<0.05 vs. CONT.

A total of 167 cases of infectious complications developed in 122 patients, and the incidence of infection per person showed increasing tendency in the three groups (ABOi, ABOi-HLAs or HLAs group) with pre-transplant PP/IVIg than in the CONT group, but it did not differ among the three groups. The incidence of postoperative bleeding was lower in the CONT group compared to the ABOi or ABOi-HLAs group (P < 0.05 for each), but it did not show any difference among the three groups with pre-transplant PP/IVIg (P > 0.05 for all) (Table 2). During the follow-up, there were 17 cases of allograft failure: 9 in the ABOi group, 2 in the HLAs group and 6 in the CONT group. The reasons for the allograft failure in the ABOi group were four patient deaths (sudden cardiac death, acute respiratory distress syndrome, sepsis, and aneurysm rupture), two cases of chronic rejection, and three cases of postoperative bleeding. The reasons for allograft failure in the HLAs group were one case of acute rejection and another case of chronic rejection. The reasons for allograft failure in the CONT group were two cases of acute rejection and three cases of chronic rejection.and one case of patient death. No allograft failure was detected in the ABOi+HLAs group. No significant difference was detected among the four groups in allograft or patient survival rates (Fig 4A and 4B). In change of allograft function, no difference was detected among the four groups up to 1 year after KT (Fig 4C).

## Discussion

In this study, we investigated the clinical outcome of ABOi+HLAs KT recipients compared with solely ABOi or HLAs recipients in terms of AAMR or overall acute rejection. We found that the outcome of the ABOi+HLAs group was similar to that of the HLAs group, and the outcome of the ABOi group was superior to that of the ABOi+HLAs and HLAs groups. This result suggests that ABOi does not have a significant impact on the outcome of KT in HLAs recipients.

First, we established the protocol for ABOi+HLAs patients in our center. Usually, a similar protocol based on the combination of RTX and PP/IVIg is used both in HLAs and ABOi KT recipients in our center and in many other centers [1,2,7,9,15,22,23,24,25,26,27,28,29]. Hence, we could apply the same desensitization protocols as those that have been used in our center for ABOi+HLAs patients. Concerning the cutoff for sufficient desensitization, a decrease of anti-A/B titer to ≤1:16 in the ABOi group and a decrease of HLA-DSA to less than the weak level (MFI <5,000) in the HLAs group have been used as criteria for KT [4,7]. Hence, in the ABOi+HLAs group, only when both an anti-A/B antibody titer of ≤1:16 and an MFI of HLA-DSA of <5000 were achieved did we consider it enough desensitization, and proceeded with transplantation. Between HLAs group and ABOi+HLAs group, only the timing of RTX infusion was different. 30 days before KT in ABOi+HLAs group and 2~3 weeks before KT in HLAs group. In both regimen, B cell depletion could be successful considering the timing of B cell depletion by RTX in previous study; hence the difference in the timing of RTX infusion may not affect the allograft outcome in two groups [30].

Next, we compared the development of AAMR between the four groups. In previous reports, including our own, transplantation across the HLA barrier resulted in higher rates of acute rejection compared with transplantation in nonsensitized patients [4,11,12,31]. In contrast, many reports showed that the acute rejection rate in ABOi KT is similar to that in ABO-compatible KT [28,29]. However, whether anti-A/B antibody has an additive impact on HLA-DSA in the development of AAMR has not been clearly determined yet. As expected, HLAs KT recipients including the ABOi+HLAs group showed a higher incidence of AAMR than the ABOi group in this study. However, the incidence of AAMR in the ABOi+HLAs group was similar to that of the HLAs group. In addition, all four AAMR cases in the ABOi+HLAs group developed with HLA-DSA and anti-A/B antibody was not detected at the time of AAMR. The above findings suggest that ABOi did not increase the risk for rejection in the HLAs KT patient group.

To further investigate the impact of anti-A/B antibody on acute rejection in HLAs KT, we performed a multivariate analysis. Previously, we found that a titer of anti-A/B antibody of ≥1:512 at baseline was an independent risk factor for acute rejection. However, HLAs and ABOi+HLAs patients were not included in that analysis; hence, we could not determine the impact of ABOi on HLAs KT patients on the basis of the result of our previous study [7]. Therefore, we included all the ABOi, HLAs, and ABOi+HLAs patients in this analysis, and finally, we found that a high baseline anti-A/B antibody titer was not a significant predictor for AAMR in this study group. In contrast, a strong HLA-DSA level at baseline was the independent risk factor for the development of AAMR, as seen in our previous study performed in patient groups not including ABOi KT [4]. This supports the idea that ABOi does not have an additive impact in the development of AAMR in the HLAs patient group.

The reason for the insignificant impact of the anti-A/B antibody in the HLAs KT group is unclear; however, it may have resulted from the lower immunogenic nature compared with the anti-HLA antibody. Indeed, the blood group Ag is composed of carbohydrate and mostly T-cell independent. In contrast, HLA Ag is composed of protein and is T-cell dependent. In addition, the anti-A/B antibody is naturally occurring, whereas anti-HLA antibodies develop as a result of exposure to foreign HLA antigens during pregnancy, blood product transfusion, or previous transplantations [14]. Owing to these differences in nature, a rebound of anti-A/B antibody after KT is less frequently observed, and a higher rate of accommodation was observed compared with HLA-DSA [7,9,14]. In contrast, rebound or *de novo* developed HLA-DSA is frequently detected even in patients without sensitization to HLA before KT, and subsequently, it is significantly associated with worse allograft outcome [3,32,33].

In the comparison of overall acute rejection, a significant increase was found in the ABOi+HLAs or HLAs group compared with the ABOi or CONT group, similarly to AAMR. The reason for the increase of not only AAMR but also total rejection is unclear; however, it is possible that activation of humoral immunity may induce T-cell-mediated immune responses [34,35]. Further investigation may be required to clarify this issue. In the comparison of infectious or postoperative bleeding complications, they showed apparent increasing tendency in the three groups with pre-transplant PP/IVIg compared to in the CONT group. But, we did not find any significant differences among those three groups. In our previous report, the strength of desensitization is significantly associated with infection, and aggressive plasma exchange was found to increase the risk for postoperative bleeding [7,8]. In ABOi, ABOi+HLAs and HLAs groups, we applied a similar desensitization protocol comprising PP/IVIg and RTX, and the number of PP/IVIg did not differ among the three groups, which resulted in a similar outcome in terms of infection and bleeding complications.

The limitation of this study is the small sample size and short-term follow-up duration. Irrespective of the higher incidence of AAMR in the ABOi+HLAs and HLAs group, no difference in allograft and patient survival rates was detected between the three groups. Study conducted in larger patients group with longer follow-up duration would make more concrete conclusion in this field. In addition, the use of different doses of RTX according to the patient’s immunologic characteristics might have caused bias. For example, we used low-dose RTX in ABOi KT patients with a low baseline anti-A/B antibody titer (≤1:128) without HLAs. On the other hand, we used typical-dose RTX in ABOi KT patients with a high baseline anti-A/B titer (≥1:512) or in HLAs irrespective of ABOi, both of which are higher-risk groups for rejection.

In conclusion, KT in patients with combined ABOi and HLAs had a similar incidence of AAMR to that in solely HLAs patients, and was inferior to that in ABOi patients. In addition, a high baseline titer of anti-A/B antibody was no longer a significant predictor for AAMR when the HLAs group was included, whereas a strong HLA-DSA at baseline was still a significant risk factor for AAMR even when the ABOi group was included. This suggests that ABOi does not have an additive immunologic risk on the clinical outcome of KT in recipients with high sensitization to HLA; hence, ABOi+HLAs KT recipients could be considered a similar risk group to HLAs KT recipients.

## References

[1] Huh KH, Kim BS, Yang J, Ahn J, Kim MG, Park JB, et al Kidney transplantation after desensitization in sensitized patients: a Korean National Audit. Int Urol Nephrol. 2012; 44: 1549–1557. 10.1007/s11255-012-0169-1 22528582

[2] Kong JM, Ahn J, Park JB, Chung BH, Yang J, Kim JK, et al ABO incompatible living donor kidney transplantation in Korea: highly uniform protocols and good medium-term outcome. Clin Transplant. 2013; 27: 875–881. 10.1111/ctr.12249 24118271

[3] Hidalgo LG, Campbell PM, Sis B, Einecke G, Mengel M, Chang J, et al De novo donor-specific antibody at the time of kidney transplant biopsy associates with microvascular pathology and late graft failure. Am J Transplant. 2009; 9: 2532–2541. 10.1111/j.1600-6143.2009.02800.x 19843031

[4] Chung BH, Choi BS, Oh EJ, Park CW, Kim JI, Moon IS, et al Clinical impact of the baseline donor-specific anti-human leukocyte antigen antibody measured by Luminex single antigen assay in living donor kidney transplant recipients after desensitization therapy. Transpl Int. 2014; 27: 49–59. 10.1111/tri.12199 24118413

[5] Toki D, Ishida H, Setoguchi K, Shimizu T, Omoto K, Shirakawa H, et al Acute antibody-mediated rejection in living ABO-incompatible kidney transplantation: long-term impact and risk factors. Am J Transplant. 2009; 9: 567–577. 10.1111/j.1600-6143.2008.02538.x 19260836

[6] Tobian AA, Shirey RS, Montgomery RA, Cai W, Haas M, Ness PM, et al ABO antibody titer and risk of antibody-mediated rejection in ABO-incompatible renal transplantation. Am J Transplant. 2010; 10: 1247–1253. 10.1111/j.1600-6143.2010.03103.x 20420632

[7] Chung BH, Lim JU, Kim Y, Kim JI, Moon IS, Choi BS, et al Impact of the baseline anti-A/B antibody titer on the clinical outcome in ABO-incompatible kidney transplantation. Nephron Clin Pract. 2013; 124: 79–88. 10.1159/000355855 24157458

[8] Chung BH, Yun JT, Ha SE, Kim JI, Moon IS, Choi BS, et al Combined use of rituximab and plasmapheresis pre-transplant increases post-transplant infections in renal transplant recipients with basiliximab induction therapy. Transpl Infect Dis. 2013; 15: 559–568. 10.1111/tid.12135 24011062

[9] Chung BH, Hong YA, Sun IO, Piao SG, Kim JI, Moon IS, et al Determination of Rituximab Dose According to Immunologic Risk in ABO-Incompatible Kidney Transplantation. Ren Fail. 2012; 34: 974–979. 10.3109/0886022X.2012.700892 22817654

[10] Chung BH, Lee JY, Kang SH, Sun IO, Choi SR, Park HS, et al Comparison of clinical outcome between high and low baseline anti-ABO antibody titers in ABO-incompatible kidney transplantation. Ren Fail. 2011; 33: 150–158. 10.3109/0886022X.2011.552149 21332336

[11] Haririan A, Nogueira J, Kukuruga D, Schweitzer E, Hess J, Gurk-Turner C, et al Positive cross-match living donor kidney transplantation: longer-term outcomes. Am J Transplant. 2009; 9: 536–542. 10.1111/j.1600-6143.2008.02524.x 19191764

[12] Marfo K, Lu A, Ling M, Akalin E Desensitization protocols and their outcome. Clin J Am Soc Nephrol. 2011; 6: 922–936. 10.2215/CJN.08140910 21441131

[13] Dean PG, Gloor JM, Stegall MD Conquering absolute contraindications to transplantation: positive-crossmatch and ABO-incompatible kidney transplantation. Surgery. 2005; 137: 269–273. 15746773 10.1016/j.surg.2004.08.002

[14] Padmanabhan A, Ratner LE, Jhang JS, Duong JK, Markowitz GS, Vasilescu ER, et al Comparative outcome analysis of ABO-incompatible and positive crossmatch renal transplantation: a single-center experience. Transplantation. 2009; 87: 1889–1896. 10.1097/TP.0b013e3181a76ae1 19543070

[15] Uchida J, Machida Y, Iwai T, Naganuma T, Kitamoto K, Iguchi T, et al Desensitization protocol in highly HLA-sensitized and ABO-incompatible high titer kidney transplantation. Transplant Proc. 2010; 42: 3998–4002. 10.1016/j.transproceed.2010.09.043 21168610

[16] Warren DS, Zachary AA, Sonnenday CJ, King KE, Cooper M, Ratner LE, et al Successful renal transplantation across simultaneous ABO incompatible and positive crossmatch barriers. Am J Transplant. 2004; 4: 561–568. 15023148 10.1111/j.1600-6143.2004.00364.x

[17] Jang JY, Kim YJ, Kim Y, Park YJ, Han K, Oh EJ Application of calculated panel reactive antibody using HLA frequencies in Koreans. Ann Lab Med. 2012; 32: 66–72. 10.3343/alm.2012.32.1.66 22259781 PMC3255493

[18] Kim Y, Yang CW, Moon IS, Kim M, Lim J, Park YJ, et al Donor-specific HLA class I and CREG antibodies in complement-dependent cytotoxicity-negative renal transplants. Ann Clin Lab Sci. 2010; 40: 330–335. 20947806

[19] Chung BH, Kim KW, Kim BM, Piao SG, Lim SW, Choi BS, et al Dysregulation of Th17 cells during the early post-transplant period in patients under calcineurin inhibitor based immunosuppression. PLoS One. 2012; 7: e42011 10.1371/journal.pone.0042011 22848688 PMC3405048

[20] Chung BH, Hong YA, Kim HG, Sun IO, Choi SR, Park HS, et al Clinical usefulness of BK virus plasma quantitative PCR to prevent BK virus associated nephropathy. Transpl Int. 2012; 25: 687–695. 10.1111/j.1432-2277.2012.01480.x 22509924

[21] Solez K, Colvin RB, Racusen LC, Haas M, Sis B, Mengel M, et al Banff 07 classification of renal allograft pathology: updates and future directions. Am J Transplant. 2008; 8: 753–760. 10.1111/j.1600-6143.2008.02159.x 18294345

[22] Gloor JM, DeGoey SR, Pineda AA, Moore SB, Prieto M, Nyberg SL, et al Overcoming a positive crossmatch in living-donor kidney transplantation. Am J Transplant. 2003; 3: 1017–1023. 12859539 10.1034/j.1600-6143.2003.00180.x

[23] Yoon HE, Hyoung BJ, Hwang HS, Lee SY, Jeon YJ, Song JC, et al Successful renal transplantation with desensitization in highly sensitized patients: a single center experience. J Korean Med Sci. 2009; 24 Suppl: S148–155. 10.3346/jkms.2009.24.S1.S148 19194545 PMC2633191

[24] Stegall MD, Gloor J, Winters JL, Moore SB, Degoey S A comparison of plasmapheresis versus high-dose IVIG desensitization in renal allograft recipients with high levels of donor specific alloantibody. Am J Transplant. 2006; 6: 346–351. 16426319 10.1111/j.1600-6143.2005.01178.x

[25] Montgomery RA, Lonze BE, King KE, Kraus ES, Kucirka LM, Locke JE, et al Desensitization in HLA-incompatible kidney recipients and survival. N Engl J Med. 2011; 365: 318–326. 10.1056/NEJMoa1012376 21793744

[26] Gloor JM, Lager DJ, Moore SB, Pineda A, Fidler ME, Larson TS, et al ABO-incompatible kidney transplantation using both A2 and non-A2 living donors. Transplantation. 2003; 75: 971–977. 12698082 10.1097/01.TP.0000058226.39732.32

[27] Montgomery JR, Berger JC, Warren DS, James NT, Montgomery RA, Segev DL Outcomes of ABO-incompatible kidney transplantation in the United States. Transplantation. 2012; 93: 603–609. 10.1097/TP.0b013e318245b2af 22290268 PMC3299822

[28] Takahashi K, Saito K, Takahara S, Okuyama A, Tanabe K, Toma H, et al Excellent long-term outcome of ABO-incompatible living donor kidney transplantation in Japan. Am J Transplant. 2004; 4: 1089–1096. 15196066 10.1111/j.1600-6143.2004.00464.x

[29] Tyden G, Donauer J, Wadstrom J, Kumlien G, Wilpert J, Nilsson T, et al Implementation of a Protocol for ABO-incompatible kidney transplantation—a three-center experience with 60 consecutive transplantations. Transplantation. 2007; 83: 1153–1155. 17496528 10.1097/01.tp.0000262570.18117.55

[30] Vieira CA, Agarwal A, Book BK, Sidner RA, Bearden CM, Gebel HM, et al Rituximab for reduction of anti-HLA antibodies in patients awaiting renal transplantation: 1. Safety, pharmacodynamics, and pharmacokinetics. Transplantation. 2004; 77: 542–548. 15084932 10.1097/01.tp.0000112934.12622.2b

[31] Caro-Oleas JL, Gonzalez-Escribano MF, Gonzalez-Roncero FM, Acevedo-Calado MJ, Cabello-Chaves V, Gentil-Govantes MA, et al Clinical relevance of HLA donor-specific antibodies detected by single antigen assay in kidney transplantation. Nephrol Dial Transplant. 2012; 27: 1231–1238. 10.1093/ndt/gfr429 21810767

[32] Everly MJ, Rebellato LM, Haisch CE, Ozawa M, Parker K, Briley KP, et al Incidence and impact of de novo donor-specific alloantibody in primary renal allografts. Transplantation. 2013; 95: 410–417. 10.1097/TP.0b013e31827d62e3 23380861

[33] Wiebe C, Gibson IW, Blydt-Hansen TD, Karpinski M, Ho J, Storsley LJ, et al Evolution and clinical pathologic correlations of de novo donor-specific HLA antibody post kidney transplant. Am J Transplant. 2012; 12: 1157–1167. 10.1111/j.1600-6143.2012.04013.x 22429309

[34] Naemi FM, Carter V, Kirby JA, Ali S Anti-donor HLA class I antibodies: pathways to endothelial cell activation and cell-mediated allograft rejection. Transplantation. 2013; 96: 258–266. 10.1097/TP.0b013e3182985504 23823649

[35] Lobo LJ, Aris RM, Schmitz J, Neuringer IP Donor-specific antibodies are associated with antibody-mediated rejection, acute cellular rejection, bronchiolitis obliterans syndrome, and cystic fibrosis after lung transplantation. J Heart Lung Transplant. 2013; 32: 70–77. 10.1016/j.healun.2012.10.007 23260706

